# Annexin A1 can inhibit the *in vitro* invasive ability of nasopharyngeal carcinoma cells possibly through Annexin A1/S100A9/Vimentin interaction

**DOI:** 10.1371/journal.pone.0174383

**Published:** 2017-03-29

**Authors:** Ying Xiao, Chenjie Ouyang, Weiguo Huang, Yunlian Tang, Weiting Fu, Ailan Cheng

**Affiliations:** 1 Cancer Research Institute, University of South China, Hengyang, China; 2 Key Laboratory of Tumor Cellular & Molecular Pathology (University of South China), College of Hunan Province, Hengyang, China; 3 Department of Pathology, Maternal and Children’s Hospital of Foshan, Foshan, Guangdong, China; 4 Hunan Province Cooperative innovation Center for Molecular Target New Drug Study, Hengyang, China; Seoul National University College of Pharmacy, REPUBLIC OF KOREA

## Abstract

Annexin A1 is a member of a large superfamily of glucocorticoid-regulated, calcium- and phospholipid-binding proteins. Our previous studies have shown that the abnormal expression of Annexin A1 is related to the occurrence and development of nasopharyngeal carcinoma (NPC). To understand the roles of Annexin A1 in the tumorigenesis of NPC, targeted proteomic analysis was performed on Annexin A1-associated proteins from NPC cells. We identified 436 proteins associated with Annexin A1, as well as two Annexin A1-interacted key proteins, S100A9 and Vimentin, which were confirmed by co-immunoprecipitation. Gene function classification revealed that the Annexin A1-associated proteins can be grouped into 21 clusters based on their molecular functions. Protein–protein interaction analysis indicated that Annexin A1 /S100A9/Vimentin interactions may be involved in the invasion and metastasis of NPC because they can form complexes in NPC cells. The down-regulation of Annexin A1 in NPC may lead to the overexpression of S100A9/Vimentin, which may increase the possibility of the invasion ability of NPC cells by adjusting the function of cytoskeleton proteins. Results suggested that the biological functions of Annexin A1 in NPC were diverse, and that Annexin A1 can inhibit the in vitro invasive ability of NPC cells through Annexin A1 /S100A9/Vimentin interaction.

## Introduction

Nasopharyngeal carcinoma (NPC) is an endemic disease that has a highly increased incidence rate (20/100,000) in Southeast Asia, Southern China, Hong Kong, and Taiwan[1–3]. It has an early tendency to spread to the local parapharyngeal space. Nodal involvement is highly frequent (70–90%) and bulky regardless of the size of the primary. Literature reports up to 11% distant metastases at present and up to 87% at autoptic studies[4], and NPC is more prone to metastasis and invasion compared with other head and neck tumors. The treatment for patients with NPC primarily relies on radiotherapy[5], but partial recurrence and distant metastasis of NPC are some of the most frequent causes of treatment failure[4]; the prognosis of NPC patient is poor. To explore the molecular mechanism and reduce the distant metastasis of NPC, the increase in the survival rate of NPC patients has been urgent concerns.

Annexin A1 (ANXA1) is the first characterized member of the Annexin superfamily and is known to bind or annex to cellular membranes in a calcium-dependent manner[6]. It is widely expressed in numerous different cell types[7]and has been involved in a broad range of molecular and cellular processes, including the maintenance of cytoskeleton and extracellular matrix integrity, tissue growth, differentiation, and inflammation[8]. In addition, considerable evidence suggests that Annexin A1 is responsible for the occurrence, development, and metastasis of NPC[6,9]. Particularly notable is the up-regulation of Annexin A1 that suppressed the proliferation, invasion, and migration of NPC cells, whereas the down-regulation of Annexin A1 promoted the proliferation, invasion and migration of NPC cells[7], suggesting that its dysregulation may play an important role in its underlying pathogenesis.

In our previous study, comparative proteomics was performed to identify differential expression proteins between the NPC and normal nasopharyngeal epithelial tissue (NNET), and the expression of Annexin A1, one of the differential proteins in NPC and NNET, was found to be involved in the metastatic potentials of NPC cell lines[10]. Annexin A1 down-regulation in NPC tissues was also significantly correlated with lymph node and distant metastasis, which suggests that Annexin A1 may play an important role in NPC metastasis[10]. However, the molecular mechanism of Annexin A1 in NPC metastasis is still unclear.

To explore the molecular mechanism of Annexin A1 in the NPC metastasis, we investigated the Annexin A1-associated proteins by targeted proteomics, including co-immunoprecipitation combined with mass spectrometry, and the biological functions of Annexin A1-associated proteins were analyzed by bioinformatics method. In addition, the correlation in the expression levels of Annexin A1 and its associated proteins S100A9 and Vimentin in different NPC cell types were evaluated; Annexin A1 /S100A9/Vimentin complex in NPC cells was detected by co-immunoprecipitation and Western blot analysis, and the effects of Annexin A1 modulation on S100A9 and Vimentin expression, as well as in vitro invasion ability of NPC cells were determined. The results indicate that Annexin A1 inhibits NPC cell invasion possibly by Annexin A1 /S100A9/Vimentin interactions.

## Materials and methods

### Reagents

Ethical approval for this investigation was obtained from the Research Ethics Committee, the University of south China of Medicine. Participants had provided their written informed consent to participate in this study, and the ethics committees had approved this consent procedure.GV146-ANXA1 and GV-102-ANXA1-RNAi plasmids and Lipofectamine 2000 were purchased from GeneChem Co., Ltd. (Shanghai, China) and Invitrogen Life Technologies, respectively. Transwell chamber and Matrigel were purchased from BD Biosciences (Franklin Lakes, NJ, USA). Bromophenol blue, EDTA, DMSO, Coomassie Brilliant Blue R-250, molecular weight marker, Tris-base, SDS, glycine, TFA, second antibodies-conjugated with horseradish peroxidase, PVDF membrane, and Protein G-Sepharose beads were purchased from GE Healthcare Life Sciences, USA. Mouse monoclonal anti-ANXA1, anti-Vimentin antibody, and IgG-antibodies were purchased from Santa Cruz Biotechnology (Santa Cruz, CA, USA). Rabbit monoclonal anti-S100A9 antibody was purchased from Abcam Biotechnology. Mercaptoethanol, iodoacetamide, and HCl were purchased from Sigma–Aldrich (St. Louis, MO, USA). All buffers were prepared using Milli-Q water.

### Cells culture

CNE, CNE1 NPC cell lines, such as 5-8F with high metastatic potential and 6-10B without metastatic potential were kindly provided by Key Laboratory of Cancer Proteomics of Chinese Ministry of Health, Xiangya Hospital, Central South University (Changsha, Hunan, China). Cells were maintained in RPMI-1640 culture medium (Hyclone, Logan, UT, USA) supplemented with 15% fetal bovine serum (FBS; Hyclone) in a humidified atmosphere of 5% CO2 at 37°C. Cells in the logarithmic growth phase were used for the experiments.

### Co-immunoprecipitation

6-10B without metastatic potential NPC cell lysates containing 600 μl (approximately 4.8 μg/μl, determined using the BCA protein assay kit, which is purchased from Beyotime Company) total protein were precleared using pre-immune serum and 60 μl Protein G agarose, shaken for 10 min, then centrifuged at 12,000 r/min for 20 min at 4°C. Thirty μl of the supernatant were carefully removed and placed into a fresh tube, then immunoprecipitated using an anti-Annexin A1 antibody (approximately 6 μg) and incubated overnight. Afterward, 60 μl 50% (dilute with PBS) Protein G agarose were added, shaken for 4 h at 4°C, then centrifuged at 12,000 r/min for 1 min at 4°C. Complexes were collected and washed three times with PBS. Complexes were eluted with 0.1 M glycine at pH 3.0 and adjusted to pH 7.5 with Tris buffer; immunoprecipitated proteins were eluted using SDS-Sample buffer containing 2-mercaptoethanol at 100°C for 3 min. Ig G displaces Annexin A1 antibody for control group. Afterward, proteins were analyzed by SDS-PAGE. After SDS–PAGE electrophoresis, proteins in the gel were detected by Coomassie Blue R-250 staining followed by in-gel trypsin digestion and mass spectrometry analysis. Proteins in the gel were transferred to PVDF membranes for Western blot analysis followed by incubation with anti-Vimentin, anti-S100A9, anti-Annexin A1 or β-actin antibody, replaced by non-immune Ig G antibody (Santa Cruz, CA; USA), which was used as negative control.

### MS and database analysis

Differential protein bands were excised carefully from the 2-D gels stained with Coomassie Brilliant Blue G-250. The destaining process consisted of two steps. First, stained gels were treated with the fresh solution (100 mmol/L NH4HCO3: 100% acetonitrile = 1:1) for 30 min, then washed twice by Milli-Q water. After washing, gel spots were dehydrated with 100% ACN for 15 min at room temperature and dried in a vacuum centrifuge. Protein samples (100 μg) were reduced with 50 mM DTT at 60°C for 1 h and then carboxyamidomethylated with 100 mM IAA at room temperature in the dark for 30 min. A proteomic grade trypsin was added to protein samples (1:20) for digestion overnight at 37°C. The tryptic peptide mixture was extracted and purified with Millipore ZipTip C18 column. Finally, four pooled samples were analyzed by 2DLC/MS/MS. Pooled mixture samples were dissolved in 500 μl SCX (25% ACN, 10 mM KH_2_PO_4_, pH 2.6) and were fractionated using HPLC column (Polysulfoethy1) from Waters. Samples were loaded onto the column in buffer A (H2O, pH = 2.6). The elution gradient was 8–27% buffer B (25% ACN, pH = 2.6; flow rate = 200 μL/min) for 60 min. Eluted peptides were collected at a rate of one fraction per minute. Each fraction was analyzed using a reverse-phase Zorbax 300SB-C18, and the elution gradient was 5–35% buffer B (0.1% formic acid, 95% ACN; flow rate, 300 nL/min for 90 min). A Triple TOF 5600 mass spectrometer was used to analyze eluted peptides from LC, and each fraction was run three times. MS data were acquired under high-sensitivity mode using the following parameters: MS/MS scans range of 400–1800 m/z per full scan, then we selected four parts with the strongest ion strength, scanning range of 100–2000 for the second time. MS/MS results, which were collected by Analyst TF will be imported to ProteinPilot ^™^ 4.5 with MGF file for database searching. Search parameters were as follows: first, the Paragon Method was selected; sample type was Identification; Cysteine alkylation was carbamoylmethylation (C); trypsin was used for digestion; instrument used was Triple TOF 5600; Special factors was selected as none; Species was Homo sapiens; ID Focus was biological modification; Database was UniProtKB/Swiss-prot FASTA (20140410); and Seach effect was Thorough ID. All the proteins which have been identified had two score: one was unused ProtScore, the other one is total ProtScore; the unused ProtScore >1.3 indicates identity or extensive homology (P< 0.05), then imported all the proteins which were indicated identity in the UniProtKB/Swiss-Prot for searching (P< 0.05). A total of 436 Annexin A1-associated proteins were analyzed by MS.

### Western blot analysis

Western blot analysis was performed as previously described[10]. In brief, immunoprecipitated complexes were separated by 10% SDS–PAGE and aliquots of cell lysates were separated using SDS-PAGE on a 12% polyacrylamide gel, then transferred to a PVDF membrane. Blots were blocked with approximately 5% nonfat dry milk for 2 h, shaken at room temperature, and then incubated with primary anti-S100A9, anti-Vimentin, anti-Annexin A1 or anti-β-actin antibody at 4°C overnight followed by incubation with a horseradish peroxidase-conjugated secondary antibody for 2 h and shaking at room temperature. Proteins were detected using an enhanced chemiluminescence Western blot system (Thermo Fisher Scientific, Inc., Waltham, MA, USA), and the band intensity was measured using densitometry combined with Image- Pro Plus 6.0 software (National Institutes of Health, Bethesda, MD, USA).

### Bioinformatics analysis

Molecular functions classification and cluster analysis were performed through GO and DAVID Bioinformatics Resources 6.7 (http://david.abcc.ncifcrf.gov/). Protein–protein interaction (PPI) analysis was done using String software (String 9.1) (http://string-db.org/newstring_cgi/show_input_page.pl)[11].

### Immunohistochemistry

The general process of tissue sections were processed was as follows: deparaffinized in turpentine, rehydrated in a graded ethanol series, and antigen retrieval solution (10 mmol/L sodium citrate buffer, pH 6.0). Sections were incubated with anti-Annexin A1 antibody (1:50 dilution), anti-S100a9 antibody (1:150 dilution), and anti-Vimentin antibody (1:50 dilution) overnight at 4°C; secondary antibodies were incubated for 30 min at 37°C on the next day. Finally, sections were incubated in 3,3-diaminobenzidine until a brown color developed and counterstained with Harris’ modified hematoxylin. As a negative control, primary antibodies were replaced with PBS. The ethical approval for this investigation was obtained from the Research Ethics Committee, the University of South China of Medicine.

A semi-quantitative analysis was used to score the staining results, in which both staining intensity and positive areas were recorded according to the methods as follows: at least 10 high-power fields were chosen randomly, and >1000 cells were counted for each section; the intensity of staining was graded on the following scale: 0, negative staining; 1, mild staining; 2, moderate staining; and 3, strong staining. The area of staining was expressed as follows: 0, <1% staining of cells in any microscopic fields; 1, 1% to 30% 2, 30% to 60% and 3, >60%. The score of extension and intensity was the final score, therefore 0 and the maximum, 6. A combined staining score (extension + intensity) of ≤2 was considered to be negative staining or low staining; a score between 3 and 4 was considered to be moderate staining; that between 5 and 6 was considered to be strong staining.

### Instant transfection

The plasmid GV146-ANXA1, GV146-ANXA1-negative, GV-102-ANXA1-RNAi, and GV102-ANXA1-RNAi-negative were purchased from GeneChem Co. (Shanghai, China). The transfection of plasmids were performed using Lipofectamine 2000 (Invitrogen), according to the manufacturer’s protocol. GV146-ANXA1-negative blank plasmids and GV146-ANXA1 recombinant plasmid were transfected into the 5-8F cells. Meanwhile, 6-10B cells were transfected with ANXA1-RNAi and ANXA1-RNAi-negative plasmids.

### Cell migration and invasion assays

The migration and invasion activities of 5-8F, 5-8F-ANXA1(-), 5-8F-ANXA1(+), 6-10B, 6-10B-RNAi(−), and 6-10B-RNAi(+) cells were assayed using a transwell cell culture chamber. Cells were added to the transwell chamber (8 μm pore size; BD Biosciences). The number of cells that migrated through the membrane was determined eight hours later. For the invasion assays, Matrigel (Matrigel:RIPA1640 = 1:6,BD Biosciences) was added to the transwell chambers and (30 μl /well) incubated overnight prior to addition of cells. The number of cells which penetrated the membrane was counted 24 h later. Migration and the invasion assays were performed in triplicate for each cell line tested.

### Wound healing assays

After transfection, 5-8F, 6-10B cells, and 5-8F 6-10B cells were added in 24-well plates at 70% confluence and cultured until they formed a monolayer that occupied 100% of the surface area. Next, a linear wound was made by scratching the monolayer with a 100 μl pipette tip and washed three times with serum-free RPMI 1640 to remove the cell debris. Cells were incubated in a humidified atmosphere of 5% CO_2_ at 37°C. Images were captured and the gap size was measured at 0 h and 24 h. The experiment was repeated three times and was analyzed by Image-Pro Plus 6.0.

### Statistical analysis

Statistical analysis was done using SPSS (version 18.0). Significant differences between two groups were compared using *t*-test in transwell cell invasion studies. Possible correlations in the expression levels of Annexin A1, S100A9 and Vimentin in NPC were analyzed using Spearman analysis. Differences were considered statistically significant for P< 0.05.

## Results

### Annexin A1-associated proteins were identified by co-immunoprecipitation and MS

**Fig 1:**
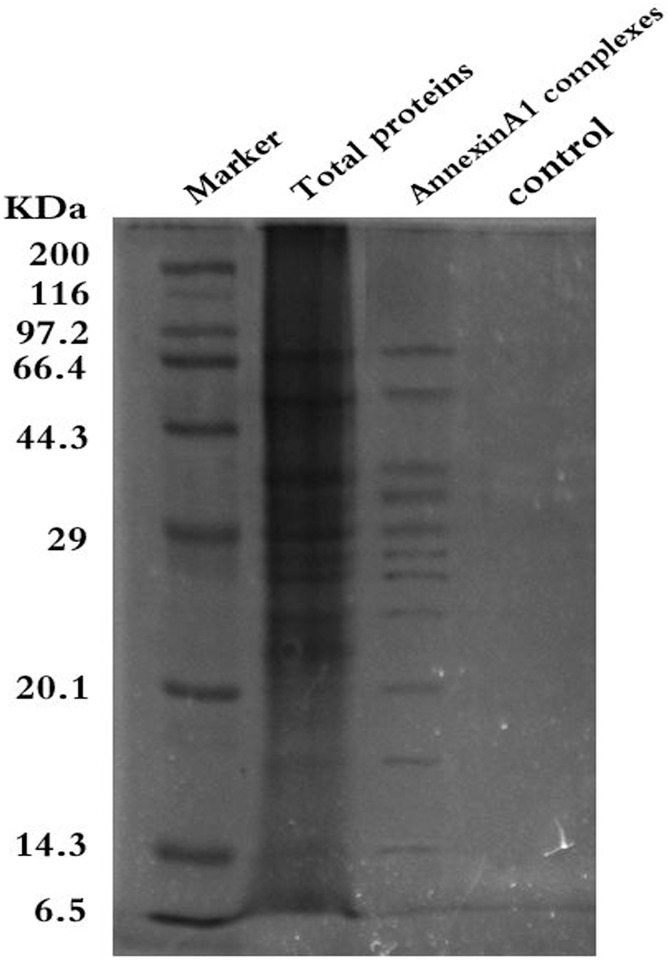
Analysis of Annexin A1 interaction in 6-10B NPC cells. Identification of Annexin A1-associated proteins by coimmunoprecipitation and MS. SDS–PAGE electrophoresis of Annexin A1 co-immunoprecipitated complex. Proteins in the gels were detected by Coomassie blue R-250 staining followed by in-geltrypsindigestion and mass spectrometric analysis. Three independent experiments were performed.

To explore proteins that interact with Annexin A1, a proteomic analysis of the Annexin A1 complexes were performed by targeted proteomics (co-immunoprecipitation combined with MS). Total proteins of NPC cell line 6-10B were collected and were subjected to co-immunoprecipitation with gel electrophoresis to separate Annexin A1 interaction proteins, as shown in Fig 1, then applied 2DLC-MS/MS to identified proteins that associated with Annexin A1 in NPC 6-10B cell. Annexin A1 antibody replaced by non-immune Ig G antibody was used as negative control. All the experiments were performed in three replicates. Based on the results, 436 proteins were identified in the Annexin A1 complex after removing proteins that were not found in the replicate experiments and removing common proteins that were found in the negative control. Then, all the identified proteins were imported into the Swiss-Prot for searching (P>0.05).

### Confirmation of the interactome of Annexin A1

**Fig 2:**
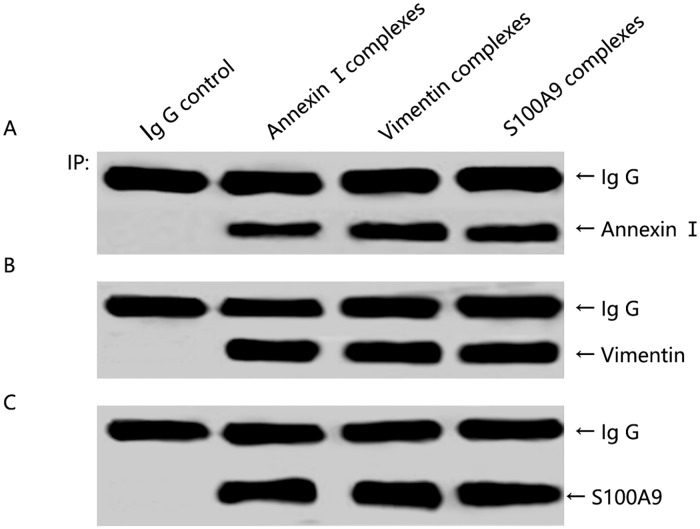
Confirmation of Annexin A1/S100A9/Vimentin interaction in 6-10B NPC cells by co-immunoprecipitation and Western blot analysis. (A) Annexin A1 can be detected in Annexin A1, S100A9 and Vimentin's co-immunoprecipitated complex by Westernblot analysis. (B) Vimentin can be detected in Annexin A1, S100A9 and Vimentin co-immunoprecipitated complex by Western blot analysis. (C) S100A9 can be detected in Annexin A1, S100A9 and Vimentin co-immunoprecipitated complex by Western blot analysis, but the three proteins cannot be detected in the non-immune Ig G control. Three independent experiments were performed.

To confirm the MS analysis results, two interesting proteins (S100A9, Vimentin) were selected and their binding was detected in the Annexin A1 in 6-10B cells by co-immunoprecipitation and Western blot analysis. As shown in Fig 2, S100A9 and Vimentin were detected in the Annexin A1 immune complex but not in the non-immune IgG control. That is, those data showed that MS analysis results are reliable for bioinformatics and PPI analysis.

### Bioinformatics analysis

A total of 436 identified Annexin A1-associated proteins were grouped into 21 functional classes by Gene Function Classification and GO analysis (including GOTERM_BP, GOTERM_MF and GOTERM_CC) based on their molecular functions (http://david.abcc.ncifcrf.gov/). Protein functions mainly involved the following: calcium-dependent phospholipid binding, signal peptide, integral to membrane, transmembrane transport, protein kinase activity, cytoplasmic vesicle part, cytoskeleton organization, etc. KEGG and Biocarta signaling pathway analyses were also performed in proteins associated with Annexin A1 in DAVID, and the results showed that Annexin-A1 interaction proteins were involved in 35 KEGG pathways and 7 Biocarta pathways. Among them, four proteins were involved in cytoskeleton signaling pathway, nine proteins in adherens junction signaling pathway, and 12 proteins win transmembrane transport signaling pathway. Some functions of proteins that were identified in the Annexin A1 complexes are closely related to the reported function of Annexin A1, such as cytoskeleton, cell cycle, and differentiation.

### Protein–protein interaction analysis

**Fig 3:**
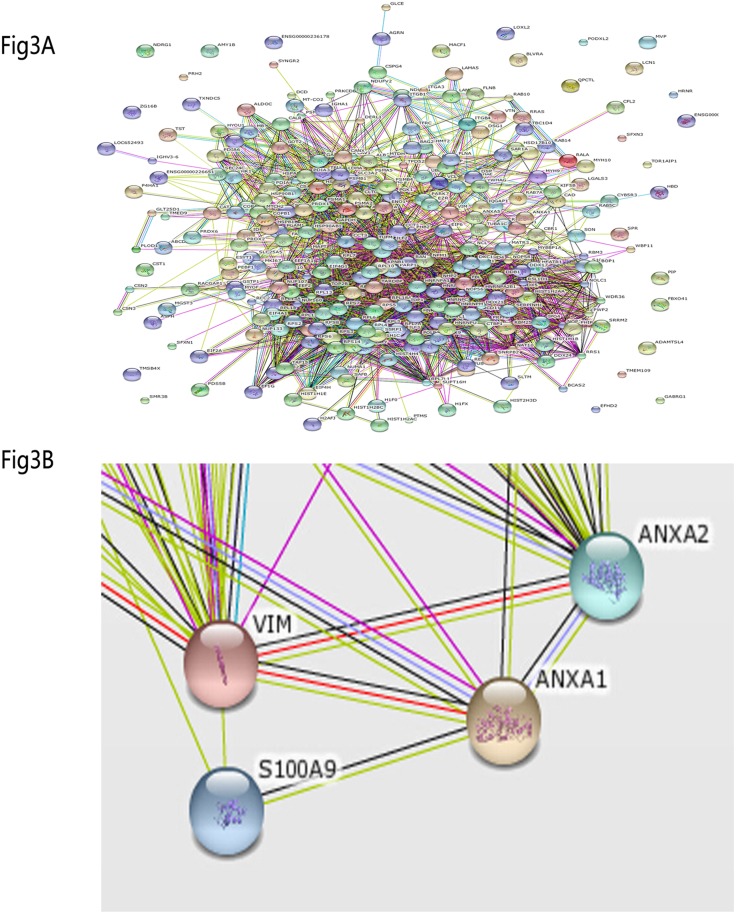
Analysis of protein–protein interaction using String 9.1(http://string-db.org/newstring_cgi/show_input_page.pl). (A) Integrated distribution network of all the interactions of Annexin A1-associated proteins is showed. Nodes show the logogram of foregone Annexin A1-associated proteins and interactions marked by lines. (B) Partial enlarged detail of interactions of Annexin A1-associated proteins. Annexin A1/S100A9/Vimentin can form complexes in NPC cells.

To identify the biological processes in proteins-associated Annexin A1 and to map the correlation of these Annexin A1-associated proteins, PPI analysis was performed using String 9.1 (http://string-db.org/newstring_cgi/show_input_page.pl). PPIs were shown in Fig 3A and 3B. Hence, Annexin A1 can interact with Vimentin and S100A9; Annexin A1 plays an important role in this PPI network. Nodes in the above-mentioned interaction maps, which are involved in calcium-dependent phospholipid blinding, signal peptide, integral to membrane, transmembrane transport, protein kinase activity, cytoplasmic vesicle part, cytoskeleton organization, may play an important role in tumorigenesis and process of NPC. Considerable evidence suggests that Annexin A1, Vimentin and S100A9 were responsible for the NPC invasion and metastasis, respectively. Our predicted interaction network shows that Annexin A1/Vimentin/S100A9 interaction tied to cytoskeletal organization. These findings suggest that Annexin A1/Vimentin/S100A9 interaction may be involved in the invasion and metastasis of NPC on theory, but have not been reported yet. Thus, we selected Annexin A1/Vimentin/S100A9 interaction for further study.

### Expression of Annexin A1, S100A9 and Vimentin in NPC tissues

**Fig 4:**
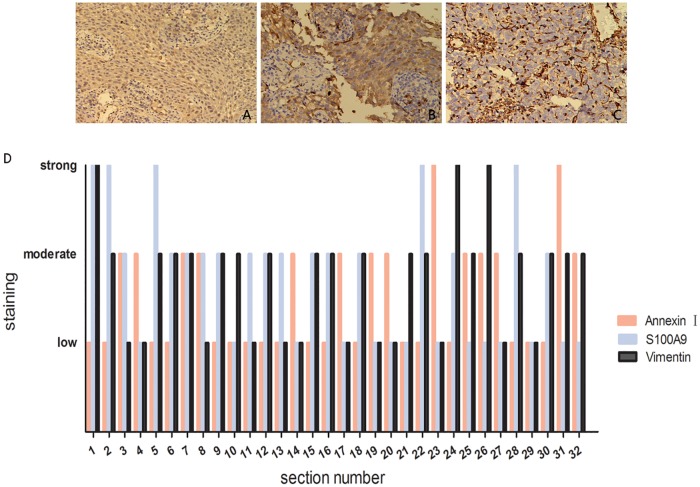
Representative results of immunohistochemistry of Annexin A1, S100A9 and Vimentin in the NPC. Immunohistochemical staining of Annexin A1(A), S100A9(B) and Vimentin (C) in the NPC tissues. Original magnification, ×200. (D) Combined staining score (extension + intensity) distribution of Annexin A1, S100A9 and Vimentin in the 32 cases of NPC tissues.

Immunohistochemistry was performed to detect the expression of Annexin A1 and its key proteins, S100A9 and Vimentin in 32 cases of NPC. The correlation in expression levels of Annexin A1, S100A9 and Vimentin in NPC was analyzed. As shown in Fig 4, S100A9 was negatively correlated with Annexin A1 expression (r = −0.640, P< 0.01); Vimentin was negatively correlated with Annexin A1 expression (r = −0.422, P< 0.05); and S100A9 was positively correlated with Vimentin expression (r = −0.390, P< 0.05). The result indicates that Annexin A1 expression is negatively correlated with the expression of S100A9 and Vimentin in NPC tissues.

### Expression of Annexin A1, Vimentin and S100A9 in different NPC cells

**Fig 5:**
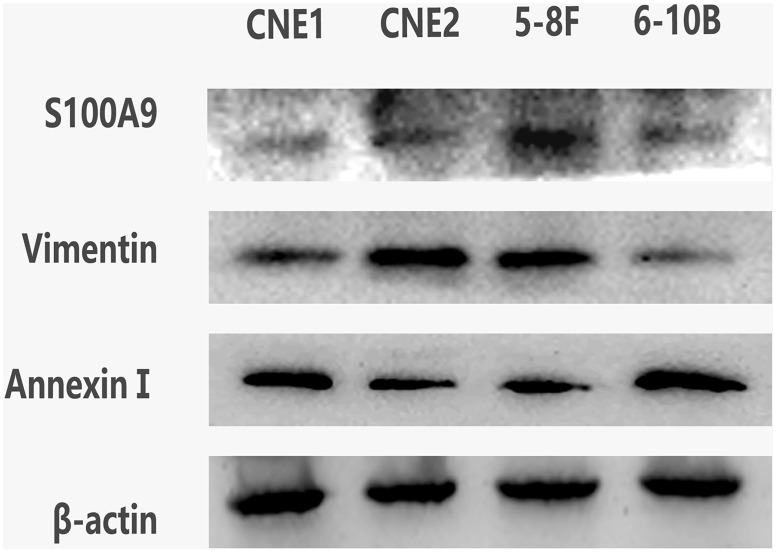
Expression of Annexin A1, Vimentin and S100A9 in different NPC cells. Western blot analysis demonstrates the expression levels of Annexin A1, Vimentin and S100A9 in the 5-8F, 6-10B, CNE1 and CNE2 cell lines. CNE1 is a highly differentiated cell line of NPC; CNE2 is a poorly differentiated cell line of NPC; 6-10B cells with low metastatic potential; 5-8F cells with high metastatic potential.β-Actin was used as an internal control for loading.

Western blot analysis was performed to detect the expression of Annexin A1 and its associated proteins Vimentin and S100A9 in CNE1, CNE2, 5-8F and 6-10B NPC cells. The correlation in expression levels of Annexin A1, Vimentin and S100A9 in NPC was analyzed. As shown in Fig 5, comparing with the CNE2 cells, which are poorly differentiated and 5-8F cells, which are of high metastatic potential, a significantly higher expression of Annexin A1 was observed, but lower expression of Vimentin and S100A9 in the CNE1 has a high differentiation, and 6-10B cells with low metastatic potential (P<0.05). The protein Annexin A1 may be inversely correlated with the metastatic potential of NPC cell lines.

### Effects of Annexin A1 modulation on the Vimentin and S100A9 expression and in vitro invasion and migration of NPC cells

**Fig 6:**
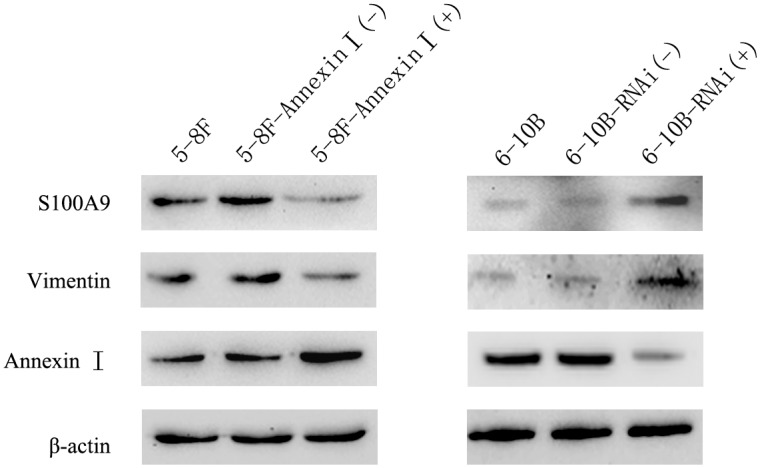
Effects of Annexin A1 modulation on the Vimentin and S100A9 expression. Western blot analysis showing the expression levels of Annexin A1, Vimentin and S100A9 in 5-8F and 6-10B NPC cells and their transfectants, 5-8F: untransfected 5-8F cells; 5-8F-Annexin A1(+): transfected 5-8F cells with Annexin A1-expressing plasmid; 5-8F-Annexin A1(−): transfected 5-8F cells with control vector; 6-10B:untransfected 6-10B cells; 6-10B siRNA(+): transfected 6-10F cells with Annexin A1 siRNA plasmid; 6-10B-siRNA(−): transfected 6-10B cells with control vector. β-Actin was used as an internal control for loading.

To study the association of Annexin A1, Vimentin and S100A9 with the NPC cells invasion ability, high metastatic 5-8F cells were immediately transfected with the GV146-ANXA1 plasmid and GV146-ANXA1-negative control plasmid and established the cell lines 5-8F-ANXA1(+) and 5-8F-ANXA1(−), respectively; non-metastatic 6-10B cells were immediately transfected with ANXA1-RNAi plasmid and ANXA1-RNAi-negative control plasmmid and established the cell lines 6-10B-siRNA(+) and 6-10B-siRNA(−), respectively. The expression of Annexin A1, Vimentin and S100A9 in the transfected and control cells were determined by Western blot analysis. As shown in Fig 6, when compared to the control, the introduction of Annexin A1 expressing vector into 5-8F cells significantly increased the Annexin A1 expression and decreased Vimentin and S100A9 expression; when compared to the control, introduction of Annexin A1-RNAi vector into 6-10B cells significantly decreased the Annexin A1 expression, and increased the Vimentin and S10A9 expression. The results suggest that Annexin A1 expression is negatively associated with the expression of Vimentin and S100A9.

**Fig 7:**
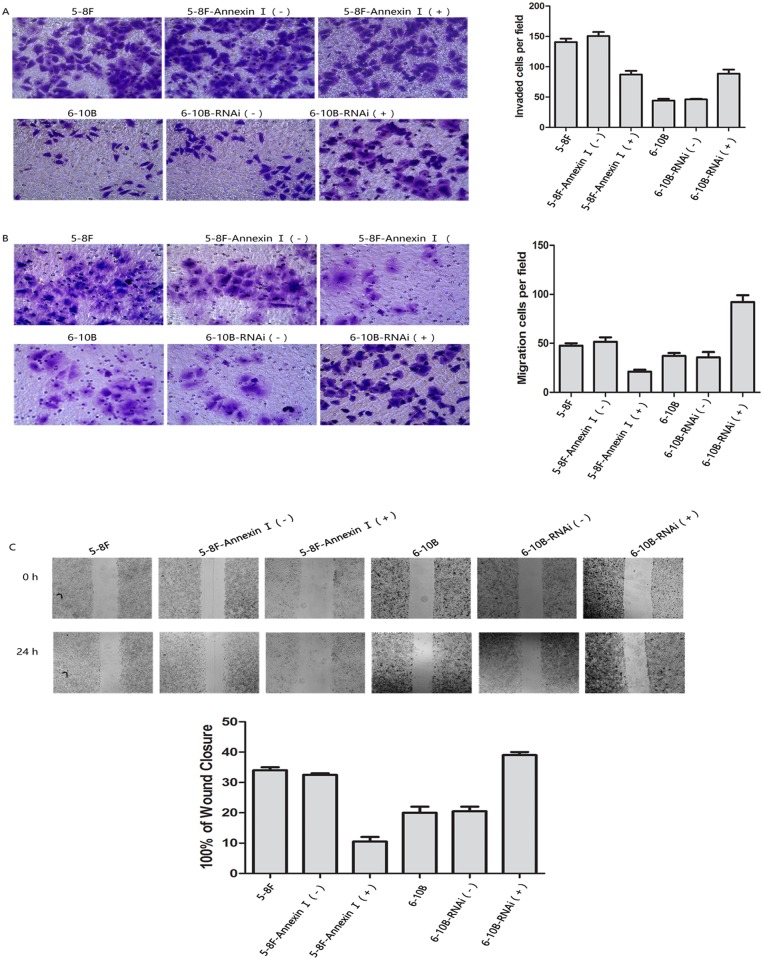
In vitro migration and invasion assays and wound healing assays. (A) In vitro migration of the 5-8F cells, 6-10B cells and their transfectants (B) In vitro invasion of the 5-8F cells, 6-10B cells and their transfectants. All the cells were measured using Transwell chambers. Bar chart showing the average number of every kind of cells which penetrated the membrane correspondingly. Original magnification, x400. (C) Wound healing assays. Data and representative images for six cell lines, as shown in the figure. Bars represent the percentage of wound closure. Original magnification, x40. Three independent experiments were performed.

We then evaluated the effect of Annexin A1 modulation on the invasion and migration of NPC cells using an in vitro invasion assay. As shown in Fig 7, invasive 5-8F cells by transfection with the Annexin A1-expressing vector were approximately 2.4-fold and 1.8-fold less than invasive 5-8F cells by transfection with the control vector (P< 0.05), and invasive 6-10B cells by transfection with the Annexin A1-RNAi vector were approximately 2.3-fold and 1.8-fold severally higher than invasive 6-10B cells by transfection with the control vector (P< 0.05). Moreover, as shown in Fig 7, wound healing assays evaluated the effect of Annexin A1 modulation on the migration of NPC cells. The results indicate that Annexin A1 expression levels are oppositely associated with the invasiveness of NPC cells in vitro.

## Discussion

Annexins are a closely related, multigene superfamily of Ca2+-regulated, phospholipid-dependent, membrane-binding proteins. Annexin A1 with a molecular mass of 37 kDa is the first known member of the Annexin superfamily[12]; it has been involved in a broad range of molecular and cellular processes, including the maintenance of cytoskeleton and extracellular matrix integrity, tissue growth, differentiation, and inflammation[8]. Moreover, considerable evidence has indicated that Annexin A1 deregulation is involved in the development, invasion, metastasis, and progression of a variety of cancers[10,13–15]. Annexin A1 was down-regulated in primary NPC, comparing primary NPC with normal nasopharyngeal epithelial tissues, and Annexin A1 was also down-regulated in LNM compared with primary NPC. Annexin A1 down-regulation is closely related to poor histological differentiation, advanced clinical stage, recurrence, poor prognosis, LNM, and distant metastasis[10]. One of the possible reasons is ANXA1 well-known ability to bind F-actin in a Ca2+-dependent mode since the protein has been found to accumulate concomitantly with the appearance of F-actin at the ruffles and at the cell–cell contacts in some biological systems[16]. Moreover, microfilaments are composed of actin polymers and a large array of actin-binding proteins[17].

S100A9 is a calcium-binding protein, which plays a prominent role in the regulation of inflammatory processes. Immune response predominantly exists as calprotectin (S100A8/A9), which has a wide plethora of intra- and extracellular functions. Intracellular functions include: facilitating the leukocyte arachidonic acid trafficking and metabolism, modulation of the tubulin-dependent cytoskeleton during migration of phagocytes, and activation of the neutrophilic NADPH-oxidase[18]. Meanwhile, S100A9 has a positive effect on the membrane aggregation of Annexin- A1 by Ca2+ collection; the membrane aggregation of Annexin A1 involves the formation of cytoskeleton, transmembrane transport, vesicle transport, and invasion[19]. Recently, more and more findings suggest that S100A9 is up-regulated in numerous cancer types, and its levels are also increased in the stroma of nasopharyngeal carcinoma[18,20].

Vimentin is a major protein of the mesenchymal intermediate filament, which maintains the cytoskeleton conformation[21]. It is referred to as the significant mark of Epithelial-mesenchymal transition (EMT), involved in the development of embryo and organ, wound tissue repair, tumor invasiveness, and metastasis[22]. EMT further plays an important role in many cancers, such as breast cancer, lung cancer, pancreatic cancer and skin squamous cell carcinoma. It is showed that Vimentin may correlated with cancer progression and prognosis at the invasive frontier in many kinds of cancers[23].

In our previous study, Annexin A1 expression was related to the metastatic potential of the NPC cell lines, and Annexin A1 down-regulation in NPC tissues was also significantly correlated with lymph node and distant metastasis (Cheng et al., 2008a, b). To understand its roles in metastasis of NPC, we further our studies to analyze Annexin A1-interacting proteins by targeted proteomics. As a result, 436 proteins associated with Annexin A1 were identified, and two Annexin A1-interacted key proteins, such as S100A9 and Vimentin were confirmed by co-immunoprecipitation and Western blot analysis. DAVID and pathway analyses showed that molecular functions of Annexin A1-associated proteins were involved in calcium-dependent phospholipid blinding, signal peptide, integral to membrane, transmembrane transport, protein kinase activity, cytoplasmic vesicle part, cytoskeleton organization, etc. Through Gene Function Classification, Annexin A1-associated proteins can be grouped into 21 clusters based on their molecular functions, and some of them were related to cytoskeleton signaling pathway, adherens junction signaling pathway, and transmembrane transport signaling pathway. The functions of some Annexin A1-associated proteins, such as the proteins which involved in cytoskeletal organization (Annexin A1, Vimentin, α-actinin 2, etc.) and vesicle transport, were closely related to the invasion and metastasis of malignant tumors.

The cytoskeleton is composed of the actin cytoskeleton, the microtubule network, and the intermediate filaments (IFs). IFs are the principal structural determinants within cells, which are composed of highly conserved globular proteins, IFs can be formed from 40 different subunit proteins which they can be distinguished into five classes, including vimentin[17]. IFs are significantly rearranged and accompanied by a significantly enhanced cell motility capacity, simultaneously, increasing Vimentin expression is its typical process[24]. S100A9 is the major calcium-binding protein of many kinds of cell. Targeted gene disruption of phagocytes reveals an essential role of this S100 protein for transendothelial migration, the molecular mechanism comprises major alterations of cytoskeletal metabolism, S100A9/ S100A8 promotes polymerization of microtubules, and S100A9 represents the regulatory subunit of S100A8/ S100A9 complexes[25,26]. Vogl Ludwig et al. observed a preferential translocation of phosphorylated S100A9 isoforms from the cytosol toward membranes and the cytoskeleton[26]. Proteins expressed in various metastatic cancers, including Ca(2+)-binding protein S100 and Annexin are used by tumor cells for plasma membrane repair (PMR), the involvement of S100, Annexin proteins and their regulation of actin cytoskeleton, leading to PMR[27]. Simultaneously, Annexin A1 protein can regulate metastasis by favoring the cell migration/invasion intracellularly, as cytoskeleton remodeling factor[28,29]. For a tumor, cells metastasize from the primary tumor; it requires disruption of cell–cell interactions from the surrounding cells and alterations of cytoskeletal, proteins of cytoskeleton are necessary for the metastasis of cancer cell, playing a key role in the invasion and metastasis of cancer. Thus, Annexin A1- associated proteins are related to the invasion and metastasis of NPC.

In this study, PPI was analyzed using STRING 9.1 software to discover how Annexin A1 interacts with its associated proteins[29]. Association of proteins can be assembled into a large network, and a network drawing can be provided by STRING, which contains both directed and undirected links. The results indicated that Annexin A1 can interact with S100A9 and Vimentin. Based on our bioinformatics analysis of Annexin A1 associated proteins and the literature on the critical roles of Annexin A1, namely, S100A9 and Vimentin in cancer invasion and metastasis, Annexin A1/Vimentin/S100A9 was selected interaction interactions for further study. Correlation analysis of the expression levels of Annexin A1, Vimentin and S100A9 in different differentiation levels(CNE1,CNE2) and in different metastatic potential(5-8F,6-10B) cells indicated that Annexin A1 expression was negatively correlated with the expression of Vimentin and S100A9, and its down-regulation may cause up-regulation of Vimentin and S100A9, which may be closely related to the metastasis of NPC. In addition, Annexin A1/Vimentin/S100A9 can form complex in NPC cells. Thus, we aimed to determine if the expression levels of Annexin A1 may affect Vimentin and S100A9 expression through Annexin A1/Vimentin/S100A9, linking to the invasion of NPC. In order to confirm our hypothesis, we established Annexin A1-up-regulated 5-8F NPC cell line and Annexin A1-down-regulated 6-10B NPC cell line and analyzed the effects of Annexin A1 modulation on the expression of Vimentin and S100A9 and in vitro NPC cell invasion. The results suggest that Annexin A1 up-regulation can decrease the expression of Vimentin and S100A9, as well as inhibit the cell invasive ability in high metastatic 5-8F cells. Moreover, Annexin A1 down-regulation can increase the expression of Vimentin and S100A9 and enhance the cell invasive ability in non-metastatic 6-10B cells. The findings suggest that Annexin A1 can inhibit the in vitro invasive ability of NPC cells possibly through Annexin A1/Vimentin/S100A9 interactions.

In conclusion, Annexin A1/Vimentin/S100A9 interaction is closely related to the invasion and metastasis of NPC. Annexin A1 down-regulation in NPC may lead to Vimentin and S100A9 overexpression, which increases the invasion ability of NPC cells possibly by activating the downstream signal molecules and reorganizing cytoskeleton.
